# Selection of the most sensitive configuration of strip array detectors for x-ray beam monitoring in radiotherapy of cancer utilizing singular value decomposition

**DOI:** 10.1007/s11517-022-02719-5

**Published:** 2022-11-24

**Authors:** Wolfgang Högele, Piotr Zygmanski

**Affiliations:** 1 Department of Computer Science and Mathematics, Munich University of Applied Sciences HM, Lothstrasse 34, 80335 Munich, Bavaria Germany; 2 Department of Radiation Oncology, Brigham and Women’s Hospital, Harvard Medical School, 75 Francis St., Boston, 02115 MA USA

**Keywords:** Multi leaf collimator, Radiotherapy, Singular values, System analysis

## Abstract

We propose a concise mathematical framework in order to compare detector configurations efficiently for x-ray beam monitoring in radiotherapy of cancer. This framework consists of the singular value decomposition (SVD) of the system matrix and the definition of an effective information threshold based on the relative error inequality utilizing the condition number of a matrix. The goal of this paper is to present the mathematical argument as well as to demonstrate its use for modeling the best detector configuration for monitoring x-ray beams in external beam therapy. This analysis depends neither on specific measurements of a given set of x-ray beams, nor does it depend in specific reconstruction algorithms of the beam shape, and therefore represents a configuration meta-analysis. In the results section, we compare three possible detector designs, each leading to a highly underdetermined system, and are able to determine their effective information content relative to each other. Furthermore, by changing design parameters, such as the geometric detector configuration, number of detectors, detector pixel size, and the x-ray beam blur, deeper insight in this challenging inverse problem is achieved and the most sensitive monitoring scheme is determined.

**Graphical Abstract:**
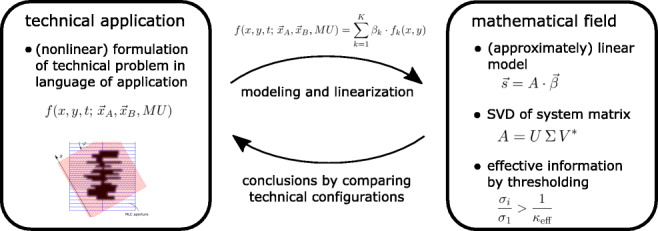
Illustration of the general approach for performing *configuration meta–analysis*.

Illustration of the general approach for performing *configuration meta–analysis*.

## Introduction

In radiotherapy of cancer with ionizing radiation, such as megavoltage x-rays produced by a medical linear accelerator, it is necessary to dynamically collimate and modulate treatment beams [19]. This is accomplished with a multileaf collimator (MLC), which is a collimator composed of many thin (≈ 2–5 mm × 8 cm × 20 cm) tungsten leaves shaping the treatment beam with submillimeter precision. The leaves are moving in and out orthogonally to the x-ray beam blocking it in some regions and allowing radiation to pass in another region as the linac irradiates tumor volume. Tumor and normal organs, which have to be spared from radiation as much as possible, have different sizes (≈ 0.5–40 cm) and shapes and therefore the motion of MLC leaves and the resulting x-ray dose can be complex. In this process, real-time monitoring and verification of MLC performance are crucial [9, 15, 27]. Development of beam monitoring detectors capable of determination of MLC motions and dose rate delivered to cancer patient in real time is of great interest to radiotherapy.

Due to technological challenges in the development and practical use to radiation detectors, several types of detector types and detection schemes have been considered: ionization chambers or diode arrays [1, 8], integral dose ionization chamber [20], flat panel detector array (TFT) [21], or high energy current (HEC) detectors [3]. During treatment, some of the x-rays interact with these radiation detectors before reaching the patient and give rise to a signal, which is converted to information about dose rate and/or MLC positions via various algorithms. The signal of the detectors arises from x-ray energy deposited inside the detector and converted to charge carriers, which are collected by a system of electrodes. The algorithms that derive clinically relevant information (MLC positions, dose rate inside detector, patient dose) depend on which features are actually measured by the specific detector and this in turn depends on the data acquisition scheme of the detector.

The question of which acquisition configuration gives rise to the most accurate estimation of dosimetric parameters in general is essential for the development of new detectors. In this study, the configuration of the detector is defined by the number of strip detector arrays [11], their orientation (angle) with respect to the x-ray beamline of the MLC collimator and with respect to the other arrays. Throughout this paper, we use the term *detector configuration* when we speak of our particular MLC monitoring device and *system configuration* when we refer to the more general mathematical framework.

In this paper, we propose and utilize a higher level mathematical *configuration meta-analysis* of different detection schemes for a specific detector type. The reason to call it a *configuration meta-analysis* is that it relies not on a specific set of shapes of x-ray fields collimated by the MLC, but on the *entirety of all possible* MLC apertures, and therefore, allows the evaluation of the system/detector configuration itself. The method we present is employing the concept of singular value decomposition (SVD), which offers a more general framework of evaluation of hypothetical detector performance for various configurations. This means, in this investigation, we are not concerned with the actual reconstruction of specific x-ray field parameters (MLC shapes) from the measured detector signals, and therefore do not rely on any specific reconstruction algorithm, but want to investigate the quality of the detector configuration itself for arbitrary x-ray fields, which is summarized in the system matrix. The system matrix does not depend on specific measurements but is a general property of the detector configuration.

System configuration comparison based directly on SVD has been applied in a few specific fields, e.g., in fluorescence diffuse optical tomography [4, 6, 13] and near-infrared tomography [26] under the name *singular value analysis* of those observation systems. It showed to be a very efficient and simple measure to utilize the number of singular values above a certain noise threshold, which are most useful for performing the tomographic reconstruction. There were also critical discussions [14], which showed that although singular value analysis is a very good overall measure for tomography setups, there are specific imaging applications for which other concepts are more helpful.

Unfortunately, the mathematical argumentation in these publications was lacking and/or the authors failed to connect the numerical results for the singular values with the general singular value properties, such as the relative error inequalities employing the condition number of the system matrix. The goal of this paper is therefore to employ the relative error inequality for a concise mathematical argument suitable for configuration meta-analysis of alternative detection schemes in beam monitoring of radiotherapy x-ray fields. The mathematical framework we propose gains deeper insight into challenging detector configuration problems which rely on the current clinical practice.

Although SVD is highly popular in many application fields, especially in data analysis combined with principal component analysis, for reconstruction algorithms based on the pseudo-inverse [24] and also for direct system analysis [5, 12], to the best of our knowledge, the full mathematical argument based on SVD and the relative error bound has not been presented for direct system configuration comparisons. We believe that this concise mathematical argumentation and the demonstration for the present challenging problem can be extended to other applications where different configurations have to be compared to each other to determine the most suitable design.

## Methods

In the following, we must distinguish between the specific *radiation device and its application to radiotherapy* from a more general *mathematical framework* that we propose for the investigation of potential design configurations of radiation devices in general. We first present a novel linear formulation of a mathematical model for a specific detector for radiotherapy beam monitoring with multiple strip detector arrays (SDA) [11]. Second, we present the essential summary of linear algebra relevant for the general singular value analysis, which allows the comparison of different linear system configurations in general (e.g., see [23] for example as a standard reference). Third, we present numerical results for the application of the general mathematical framework to the specific radiotherapy beam monitoring device (SDA). Finally, we provide a critical discussion of the proposed mathematical method and its most salient features as well as possible other uses of the method to radiotherapy problems requiring comparison of different design configurations of instrumentation.

### Mathematical formulation of MLC QA measurements in radiotherapy

#### Application and detector

During cancer patient treatment, a medical linac radiates a high-energy megavoltage photon beam via a delivery system composed of a rotating linac gantry with a rotating dynamic multileaf collimator (MLC). The patient is lying on a robotic treatment table. The MLC of a medical linac is composed of typically 120 thin leaves and is able to shape this photon beam right at the exit of the gantry head. Based on the clinical target and organs at risk, a radiation plan is generated which exactly defines the motion of the rotating medical linac as well as all the MLC positions at each time point. Furthermore, the beam intensity is also modulated by the monitor units (MUs) for each MLC segment. All treatment parameters are used in the treatment planing system (TPS) to compute the dose in the patient and are exported to the linac for delivery of the treatment.

The clinical parameters (e.g., the MLC leaf positions and the MU) in the TPS constitute the ideal or the reference for the actual delivery. Thus, a detector that monitors the actual treatment delivery measures signals $\vec {s}$ from which the clinical parameters are derived and provides deviations from the ideal TPS parameters. The deviations between these MLC/MU parameters lead to dose differences in the patient anatomy. In the present paper, we limit the work to MLC leaf positions and MU fraction per segment as clinical parameters (i.e., typically 121 free parameters) which are hypothetically determined by a specific detector that has a number of possible embodiments (configurations). The purpose in this paper is not to determine the actual values of *particular clinical beams* (as for example performed in [11]) but the efficacy of each detector configuration to provide a certain level of accuracy in determination of the clinical parameters for *arbitrary clinical beams*.

**Fig. 1:**
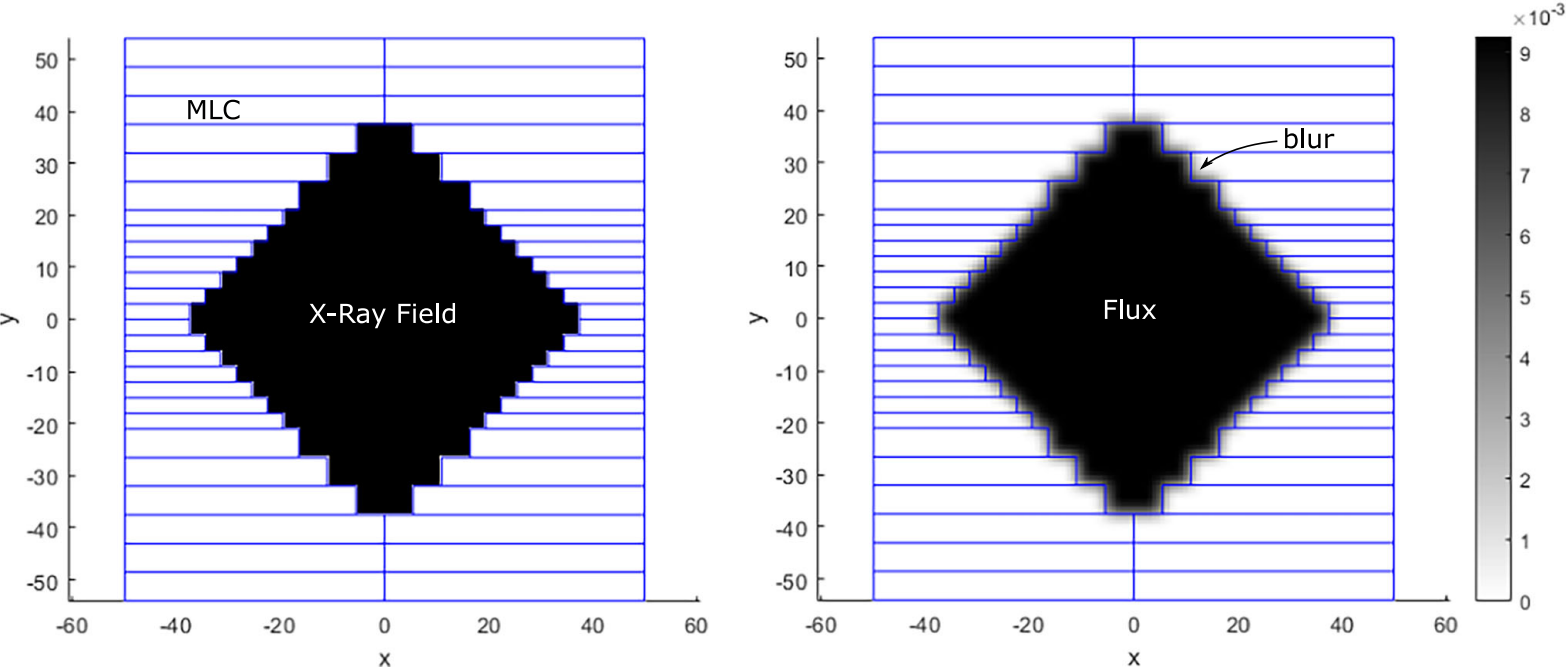
Schematic demonstration of a MLC aperture configuration and the true leaf positions as blue lines. Left: the binary model representing the MLC aperture without any dose model, Right: a very simplistic dose model applying a 2D gaussian blur representing $f(x,y,t; \vec {x}_{A},\vec {x}_{B},MU)$

The detector configuration is presented and discussed in the subsequent Section 2.1.6. The detector is mounted on the linac below the MLC. The MLC is composed of two leaf banks (left and right), and each bank typically consists of 60 parallel leaves aligned along *x*-axis (Fig. 1). The leaves move and form MLC segments that are delivered with a certain amount of radiation controlled by monitor unit fraction.

The detector configuration considered here is the following: The detector consists of parallel strip detectors and each strip detector measures dose independently as an integral dose along the whole length of the strip. The strip array covers the whole irradiation region plus extra margins. Therefore, the strip detector array measures projected/cumulative doses. The strip detector array can be oriented a priori at any angle with respect to the MLC. However, in this paper, we consider only 3 possible angles, which have certain motivation behind: 0^∘^ (along *x*-axis), 90^∘^ (along *y*-axis), 45^∘^/135^∘^ (diagonal). A similar detector design was studied in reference [2] using a different mathematical framework.

#### X-ray generated treatment fields

For each point in time, there is a well-defined MLC shape (aperture) collimated by typically 60 leaves on the left and 60 leaves on the right bank, leading to typically 120 unknown parameters. For each MLC segment, there is a certain dose delivery at constant rate, referred to as MU fraction. Based on the known MLC geometry as well as the given MUs, the medical linac will generate a 2D flux or fluence field, *f*(*x*,*y*,*t*) where *x* is defined to be parallel to the leaf pair motions, *y* is defined to be orthogonal, and *t* is time. The flux *f*(*x*,*y*,*t*) depends on the leaf pair positions, which we will denote as $\vec {x}_{A}$ and $\vec {x}_{B}$ for the left and right MLC leaves, and the MU, which we will denote as *MU*, leading to the explicit description of dependencies $f(x,y,t; \vec {x}_{A},\vec {x}_{B},MU)$.

We assume that based on a beam model we can sufficiently accurately calculate the 2D fluence field, given the leaf pair positions as well as the MUs. For the investigations at hand, we will discuss different dose models from very unrealisitic to very realistic ones in order to investigate the dependence of the inverse problem with respect to the modelling (see Fig. 1 for examples of the 2D fluence field).

At first, we realize that the original fluence field $f(x,y,t; \vec {x}_{A},\vec {x}_{B},MU)$ is a nonlinear function with regard to the leaf position, but it is linear with regard to MU. This can be directly seen by the violation of the scalar multiplication (for a given $\gamma \in \mathbb {R}$):

$$ f(x,y,t; \gamma\cdot\vec{x}_{A},\vec{x}_{B},MU) \neq \gamma\cdot f(x,y,t; \vec{x}_{A},\vec{x}_{B},MU) , $$

since the field intensity is not uniformly changed only if a subset of MLC leave positions is locally scaled. For this investigation of the optimal geometry, we try to find an approximate linear presentation of all possible 2D fluence fields that can be shaped by the original field parameters $\vec {x}_{A},\vec {x}_{B},MU$.

#### X-ray beam model

The portion of the flux that is stopped by the detector gives rise to detector signal which can be computed by convolution of the MLC aperture and Gaussian dose deposition kernels [16–18]. Since each MLC segment is composed of small rectangular shapes corresponding to each open MLC leaf pair, this gives rise to a sum of beamlets. Such a beamlet representation of dose can also be used for computation of dose in patients and mathematically it represents superposition-convolution dose models when the heterogeneities associated with the patient anatomy are properly accounted for in the model. In our case, the detector is homogeneous and thin and thus the Gaussian convolution model is sufficient [22].

A useful representation in order to describe all possible field shapes is the pencil beam approach: All fluence fields can be approximated by the weighted sum of pencil beams. We utilize here singular pencil beams of 1-mm width and the corresponding leaf pair height. Since the blur of the flux can be well approximated by a Gaussian convolution model, this is not affecting the linearity of the fluence field. Such a beam model can be extended to slightly different blur magnitudes for different layers in overlapping detector geometries. It is pointed out that this would not change the theory or application of the detector comparison approach in this paper.

Utilizing the pencil beams for all leaf pairs (e.g., with a practically useful number of 45 leaf pairs) and 1-mm gap (e.g., 150 pencil beams per leaf pair, corresponding to a 15 cm maximum leaf pair opening), we get in total *K* = 43 × 150 = 6750 individual pencil beams *f*_*k*_(*x*,*y*) ($k=1,\dots ,K$). Then we can approximate the true fluence field by

$$ f(x,y,t; \vec{x}_{A},\vec{x}_{B},MU) = \sum\limits_{k=1}^{K} \beta_{k}\cdot f_{k}(x,y) $$

when still neglecting the time dependency of the field shape. We get *K* parameters *β*_*k*_ which are the weights of the individual pencil beams that approximate the real the fluence field shape. It is pointed out that the clinical relevant parameters $\vec {x}_{A},\vec {x}_{B},MU$ can only indirectly be determined by the parameters *β*_*k*_. Obviously, this is only an approximation to real fluence fields, since

1. due to the discretization to 1-mm gaps, we cannot perfectly generate submillimeter MLC leaf positions (this can actually be resolved by utilizing smaller gap sizes and will be done in the results section), and
2. with this linear representation, we can generate many more fluence fields than are actually possible. For example, in reality, MLC leaf pairs are open or not — in this representation, we could generate every millimeter another totally arbitrary intensity which corresponds to the huge amount of degrees of freedom.

Despite of these differences between the true fluence fields and this linear representation, we can still summarize: All real field shapes can be described with minor differences with such a pencil beam approximation.

#### The forward problem of measurement

For the proposed general setup of this paper, we assume to have one or several 1D detectors, which are directly mounted to the MLC frame. This means that even if the MLC is rotating around itself, the detector will rotate accordingly. This means for the detector, the *x*- and *y*-directions are always the same as the *x*- and *y*-directions of the MLC.

Furthermore, we consider narrow strip line detectors, i.e., each of those detectors is providing a 1D signal (we call projections). The strip detectors are thin along the beam line and narrow/long across the beam line. Essentially, each detector pixel collects all the intensities of the 2D fluence field along a line integral. The line detector is at a fixed angle *φ* relative to the fluence field. Furthermore, let us say, the line detector has a 1D pixel resolution of Δ*b* and *N* pixels. Then for pixel *n* the collecting line (the path over which the integration occurs) can be written as ($u\in \mathbb {R}$) (see [11])

$$ \vec{l}_{n}(u) = u\cdot\left( \begin{array}{c} \cos(\varphi)\\ \sin(\varphi) \end{array}\right) +\left( n - \frac{N}{2}\right)\cdot{\Delta} b \left( \begin{array}{c} -\sin(\varphi)\\ \cos(\varphi) \end{array}\right) . $$

We assume that the strip detector array is oriented at some fixed angle *φ* with respect to the MLC coordinate system. The detector signal *s* at pixel *n* can then be calculated as (see [11])

$$ s_{n} = \int\limits_{-\infty}^{\infty} f(l_{n,1}(u),l_{n,2}(u),t; \vec{x}_{A},\vec{x}_{B},MU) \text{d}u . $$

**Fig. 2:**
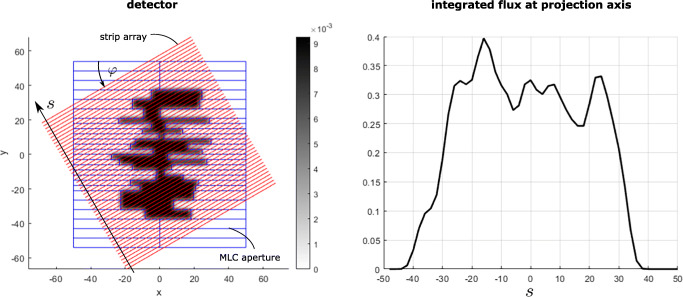
Schematic illustration of the signal generation by the proposed detector type. Left: Examples of integral paths of the 1D detector strip array (red lines) and the projection axis *s*, with an angle of *φ* = 30^∘^ and *N* = 50 pixels. Right: The corresponding signal collected by the strip array detector

For example, see Fig. 2 for an illustration of the integral paths of one strip array detector and the resulting detector signal.

#### Derivation of the system matrix based on the forward problem

When inserting the 2D fluence field decomposition to the forward problem, we get for every detector pixel *n*

$$ \begin{array}{@{}rcl@{}} s_{n} &=& \int\limits_{-\infty}^{\infty} f(l_{n,1}(u),l_{n,2}(u),t; \vec{x}_{A},\vec{x}_{B},MU) \text{d}u \\ &=& \int\limits_{-\infty}^{\infty} \sum\limits_{k=1}^{K} \beta_{k}\cdot f_{k}(l_{n,1}(u),l_{n,2}(u)) \text{d}u \\ &=& \sum\limits_{k=1}^{K} \beta_{k}\cdot \int\limits_{-\infty}^{\infty} f_{k}(l_{n,1}(u),l_{n,2}(u)) \text{d}u , \end{array} $$

and when calculating the full 1D detector signal for all *N* pixels, we get

$$ \vec{s} = \sum\limits_{k=1}^{K} \beta_{k}\cdot \left( \begin{array}{c} \vdots\\ \int\limits_{-\infty}^{\infty} f_{k}(l_{n,1}(u),l_{n,2}(u)) \text{d}u\\ \vdots \end{array}\right) , $$

with the detector signal vector $\vec {s}\in \mathbb {R}^{N}$. Looking at it as a linear combination of vectors, we can further rewrite this directly into the matrix equation

$$ \begin{array}{@{}rcl@{}} \vec{s} &=& \left( \begin{array}{ccc} {\vdots} & {\dots} & \vdots\\ \int\limits_{-\infty}^{\infty} f_{1}(l_{n,1}(u),l_{n,2}(u)) \text{d}u & {\dots} & \int\limits_{-\infty}^{\infty} f_{K}(l_{n,1}(u),l_{n,2}(u)) \text{d}u\\ {\vdots} & {\dots} & \vdots \end{array}\right) \\ && \cdot \left( \begin{array}{c} \beta_{1} \\ {\vdots} \\ \beta_{K} \end{array}\right) = D\cdot \vec{\beta} , \end{array} $$

with the projecting matrix $D\in \mathbb {R}^{N\times K}$ and vector of the degrees of freedom of the field $\vec {\beta }\in \mathbb {R}^{K}$. Please note, in every column of this matrix *D*, the detector signal of the corresponding single pencil beam is stored.

If we not only have one detector, but many, say *M*, detectors then this matrix equation still holds, but for the corresponding line integral paths described by $\vec {l}_{n}$ we need to show the dependency on detector orientation *φ*_*m*_ and rewrite this to $\vec {l}_{m,n}$. This leads to the *system matrix equation* of the measurement which consists of the stacking of the linear equations of each individual detector under each other:

$$ \begin{array}{@{}rcl@{}} \left( \begin{array}{c} \vec{s}_{1}\\ {\vdots} \\ \vec{s}_{M} \end{array}\right) &=& \left( \begin{array}{ccc} {\vdots} & {\dots} & \vdots\\ \int\limits_{-\infty}^{\infty} f_{1}(l_{1,n,1}(u),l_{1,n,2}(u)) \text{d}u & {\dots} & \int\limits_{-\infty}^{\infty} f_{K}(l_{1,n,1}(u),l_{1,n,2}(u)) \text{d}u\\ {\vdots} & {\dots} & \vdots\\ \int\limits_{-\infty}^{\infty} f_{1}(l_{M,n,1}(u),l_{M,n,2}(u)) \text{d}u & {\dots} & \int\limits_{-\infty}^{\infty} f_{K}(l_{M,n,1}(u),l_{M,n,2}(u)) \text{d}u\\ {\vdots} & {\dots} & \vdots \end{array}\right) \cdot \vec{\beta}\\ &= &\left( \begin{array}{c} D_{1}\\ {\vdots} \\ D_{M} \end{array}\right) \cdot \vec{\beta}\\ &=& A \cdot \vec{\beta} , \end{array} $$

with the left hand side vector being element of $\mathbb {R}^{M\cdot N}$ as the vector of all measured detector signals, $A\in \mathbb {R}^{M\cdot N\times K}$ the system matrix of the detector signal generation and $\vec {\beta }\in \mathbb {R}^{K}$ the degrees of freedom that describe all possible fluence field shapes.

#### Specific detector configurations

**Fig. 3:**
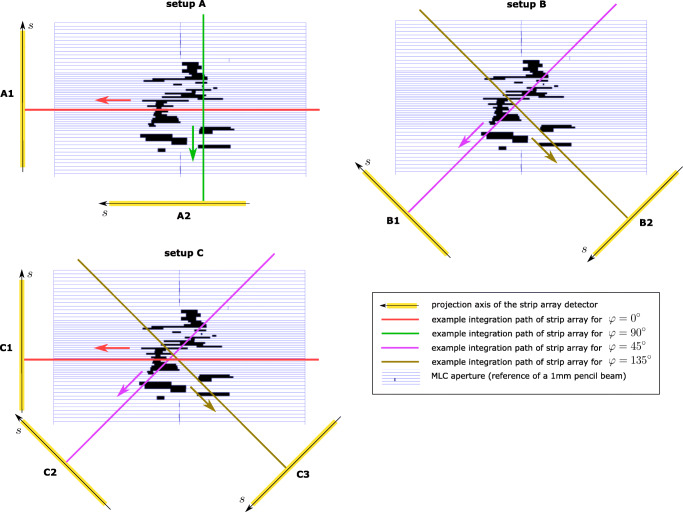
Illustration of the three different detector configurations with an example beam MLC aperture. In addition to the detector geometry relative to the MLC, the integration path and the projection axis of the strip array detector are presented. Top left: Setup A with two orthogonal detectors which are aligned to the MLC orientation (*φ* = 0^∘^ (A1) and 90^∘^ (A2)), Top right: Setup B with two orthogonal detectors which are rotated by 45^∘^ relative to the MLC orientation (*φ* = 45^∘^ (B1) and 135^∘^ (B2)). Bottom: Setup C which is setup B with an additional detector which is orthogonal to the MLC leaf pair motion (*φ* = 0^∘^ (C1), 45^∘^ (C2), and 135^∘^ (C3))

In order to demonstrate the potential of the system configuration comparison, we will utilize three different detector configurations shown in Fig. 3, with two configurations with two detectors (setup A and B) and one configuration with three detectors (setup C).

### The general singular value analysis process

We now introduce the general mathematical framework for performing the configuration meta-analysis based on singular value analysis applied to linear observation equations. We use the more general term *system configuration* in order to highlight the generality of this approach.

#### SVD of an observational system matrix

In a detection scenario, we assume to have a linear or approximately linear relationship between parameters $\vec {\beta }\in \mathbb {R}^{K}$ we want to learn about and the actually observed signals $\vec {s}\in \mathbb {R}^{N}$:

$$ \vec{s} = A\cdot\vec{\beta} $$

with the system matrix of the observation $A\in \mathbb {R}^{N\times K}$, such as in the presented mathematical formulation of the MLC QA measurements with strip detector arrays. Such equations appear, e.g., directly in static observations (e.g., see the explanatory example in Appendix ??), as an observation equation in stochastic dynamic Kalman filters [23], or as in our case: as a linearization of a nonlinear observation equation, see [7] for an example in control theory. In many practical purposes, we typically have *N* ≥ *K* and a system matrix *A* which has full column rank. In this situation, one can reconstruct the parameters $\vec {\beta }$ directly from observations $\vec {s}$ utilizing the pseudo-inverse or similar methods.

In this investigation, we are not concerned with the actual reconstruction of $\vec {\beta }$ but want to investigate the quality of the measurement configuration itself which is summarized in the system matrix (without measurements). Therefore, we can focus also on completely non-sufficient observation scenarios. Indeed, in the application, we will present a case with *N* << *K*.

When applying the singular value decomposition (SVD) to *A*, as a partial result, we get the sorted list of the singular values $\sigma _{1}\geq \sigma _{2}\geq \dots \geq \sigma _{r}$ and the residual list of zeros $\sigma _{r+1}=\sigma _{\min \limits (N,K)} = 0$. In this nomenclature, *r* represents the *rank of matrix* *A*. A more detailed description of the SVD is presented in the Appendix ??. The singular values contain relevant information about *A*, e.g., the scaling of coordinates that a vector $\vec {\beta }$ undergoes when applied to $A\cdot \vec {\beta }$.

#### Condition number of the system matrix vs. measurement noise

In general, the condition number of a matrix *A* is defined as

$$ \kappa(A) = \frac{\sigma_{1}}{\sigma_{r}} $$

with *σ*_1_ the largest and *σ*_*r*_ the smallest singular value greater than 0. A very well-known fact of numerical linear algebra is: If the measurement $\vec {s}$ has an additive error $\vec {\varepsilon }$, maybe due to measurement noise or data precision, we get a relative error bound of the solution [23]

$$ \frac{\| {\Delta} \vec{\beta} \|}{\| \vec{\beta} \|} \leq \kappa(A)\cdot\frac{\| \vec{\varepsilon} \|}{\| \vec{s} \|} = \frac{\sigma_{1}}{\sigma_{r}}\cdot\frac{\| \vec{\varepsilon} \|}{\| \vec{s} \|} , $$

which can be read in the following way: If there is a given relative measurement error $\frac {\|\vec {\varepsilon }\|}{\|\vec {s}\|}$ (which can be directly related to the signal-to-noise ratio (SNR) of the measurement) and we still want to have a worst case relative error in the result of $\frac {\| {\Delta } \vec {\beta } \|}{\| \vec {\beta } \|}$ then — according to this upper bound — we would be allowed to have a condition number *κ*_eff_ for the sharpest bound (*κ*_eff_ ≥ 1). Although it is not necessary in our investigation, one can always modify the real system matrix *A* in order to meet this condition number approximately, i.e., *κ*(*A*) ≈ *κ*_eff_, by utilizing the corresponding *low rank approximation* [23].

Clearly, this is an upper estimation for the maximum relative error estimating for all possible $\vec {s}$ and $\vec {\varepsilon }$. In real measurement scenarios, typically this limit is not achieved since the vectors $\vec {\varepsilon }$ and $\vec {s}$ would need to point in very specific directions (see the explanatory example in Appendix ?? and the Appendix ?? for a detailed discussion on this).

To give a numerical example, a relative measurement error $\frac {\|\vec {\varepsilon }\|}{\|\vec {s}\|}=10^{-2}$ and a worst case relative solution error of $\frac {\| {\Delta } \vec {\beta } \|}{\| \vec {\beta } \|} =10^{-1}$ (which means a relative error of ± 10*%*) would simply allow for a condition number of *κ*_eff_ = 10 for the sharpest bound. With respect to the practically small probability of the worst case in this upper error bound, in a very pragmatic way, one could even risk to allow a maximum relative error of $\frac {\| {\Delta } \vec {\beta } \|}{\| \vec {\beta } \|} = 1$ (i.e., at worst relative errors of the solution up to ± 100*%*), which would then lead to *κ*_eff_ = 100.

#### Effective information threshold

We have on the one side, the system matrix *A* and its singular values, on the other side, we get (by looking at the maximum allowed relative measurement and solution errors) a desired condition number *κ*_eff_ to fulfill the relative error bound sharply.

At this point, it is important to conclude, independently to which one sets these relative errors and what the original *κ*(*A*) of the system matrix is, there is an *effective information threshold* for all singular values of *A*, which must fulfill

$$ \frac{\sigma_{i}}{\sigma_{1}} > \frac{1}{\kappa_{\text{eff}}} (i=1,\dots,r) , $$

in order to meet these criteria sharply. The number of singular values that fulfill this *effective information threshold* represents the *effectively* usable number of linearily independent equations in the system matrix (i.e., an effective rank with respect to the magnitude of the measurement noise). For the practical extraction of these corresponding equations, one needs to apply the reduced rank approximation of *A* where all singular values not fulfilling this bound are set to zero.

By working with 1/*κ*_eff_ as a threshold, we essentially are focussing on thresholding normalized singular value distributions $\frac {\sigma _{i}}{\sigma _{1}}$ ($i=1,\dots ,r$) for comparison of different system configurations and not on thresholding the absolute (not normalized) singular values [4, 6, 13, 26].

**Fig. 4:**
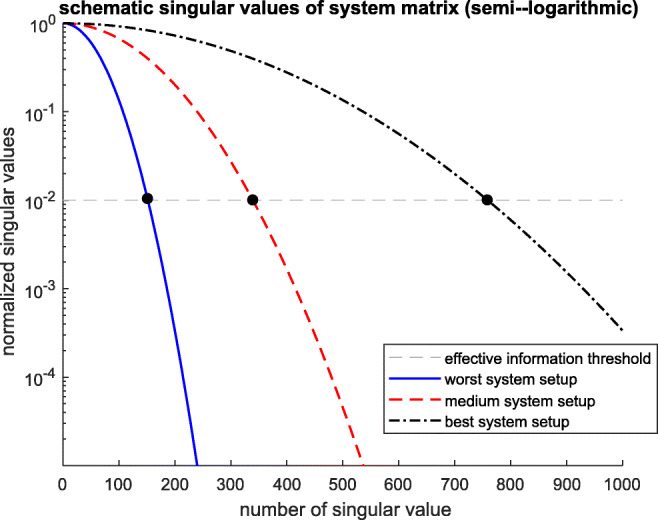
Schematic examples of the normalized singular values plot with semi-logarithmic scaling. Three system configurations are compared relative to the effective information threshold (horizontal dashed line). The number of singular values above the effective information threshold are the fewest for the worst system (continuous line) and largest for the best system (dash-dotted line)

What does this tell us about comparing different system/ detector configurations and their information content? Quite straightforwardly, the system with *more singular values* above this *effective information threshold* does contain more information about $\vec {\beta }$ than the system that has a smaller number of above-threshold values. Based on this number, different system configurations can be directly compared and optimized. This comparison can be done quickly in an overview plot of the normalized singular values with the effective information threshold as in Fig. 4. The further the intersection of the normalized singular values with the effective information threshold is to the right, the more effective information is contained in the system configuration.

#### Summarizing the singular value analysis process

This framework can be summarized to the following algorithm:

1. Model the specific observation/detection scheme in a specific application by expressing it in terms of linear equations $\vec {s} = A\cdot \vec {\beta }$ where $\vec {s}$ represent the measurements and $\vec {\beta }$ are the unknown parameters.
2. Define the expected level of relative measurement error $\frac {\|\vec {\varepsilon }\|}{\|\vec {s}\|}$ by assessing the measurement device. This can be the typical or maximal error magnitudes, depending on the perspective in the application.
3. Define the desired or target relative solution error $\frac {\| {\Delta } \vec {\beta } \|}{\| \vec {\beta } \|}$ for the application (keeping in mind that this could a small or a large number, even ≈ 1 in some applications, see the discussion in the Appendix ??).
4. Calculate the condition number *κ*_eff_ corresponding to the sharpest bound with
  $$ \kappa_{\text{eff}} = \left( \frac{\|\vec{s}\|}{\|\vec{\varepsilon}\|} \right)\cdot\left( \frac{\| {\Delta} \vec{\beta} \|}{\| \vec{\beta} \|}\right) $$
5. Perform the SVD of *A* and count the number of singular values above the *effective information threshold*
  $$ \frac{\sigma_{i}}{\sigma_{1}} > \frac{1}{\kappa_{\text{eff}}} (i=1,\dots,r) . $$
6. Repeating steps 1–5 for several competing system configurations, one can directly compare them. The system with the highest number of singular values above the *effective information threshold* overall contains the most information about $\vec {\beta }$.
  In order to judge the differences between different configurations, one can directly set the number of singular values above this threshold relative to the number of unknown parameters (i.e., the number of entries of $\vec {\beta }$). As an extreme case, if this ratio achieves 1 (i.e., 100*%*), then one can completely reconstruct $\vec {\beta }$ based on inverting the linear system of equations even with the presence of a relative measurement error.

We highly recommend the reader to the explanatory example in the Appendix ?? in order to gain a straightforward understanding of this approach for a simple reconstruction problem.

## Results

### Comparison of specific detection configurations

When looking at the linear system of equations, one realizes quickly, that for example for *M* = 3 detectors with a pixel resolution of *N* = 400 pixels per detector (corresponding to a pixel width of 0.75 mm) and *K* = 6750 pencil beams we would get an observational system matrix *A* of shape *M* ⋅ *N* × *K* = 1200 × 6750, which is an underdetermined system. If we would want to solve the inverse problem with this equation, we definitely would need to incorporate additional information, such as that not every millimeter a totally different intensity could occur. For this specific investigation of comparing different detector geometries, this is actually not very important. Because the system matrix *A* itself tells us how much information we gather by given detector geometries relative to each other as described in the general theory section.

**Fig. 5:**
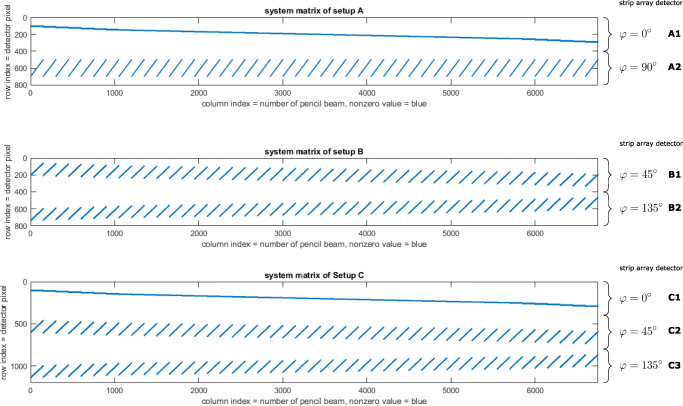
Illustration of the system matrices for setup A (top), B (center), and C (bottom). Blue areas correspond to nonzero entries in the matrix, all other entries are zero. Rows that are always zero correspond to detector pixels outside the MLC aperture area covered by the pencil beams

The system matrices for the three detector configurations (setup A and B: 800 × 6750, setup C: 1200 × 6750) are shown as maps (blue: nonzero value, white: zero) in Fig. 5. Each column of each system matrix consists of the detection signal for each pencil beam. This means, for setup A and B, we have two signal spots in each column, since we have two detectors stacked under each other, and for setup C, we have three signals. Going the columns of the system matrix to the right, we change the individual pencil beam, until all possible pencil beam signals are covered by the system matrix. The order of how we go through the pencil beams generates the characteristic patterns in Fig. 5. In consequence, the system matrix can be viewed as the pencil beam representation in the detector configuration.

**Fig. 6:**
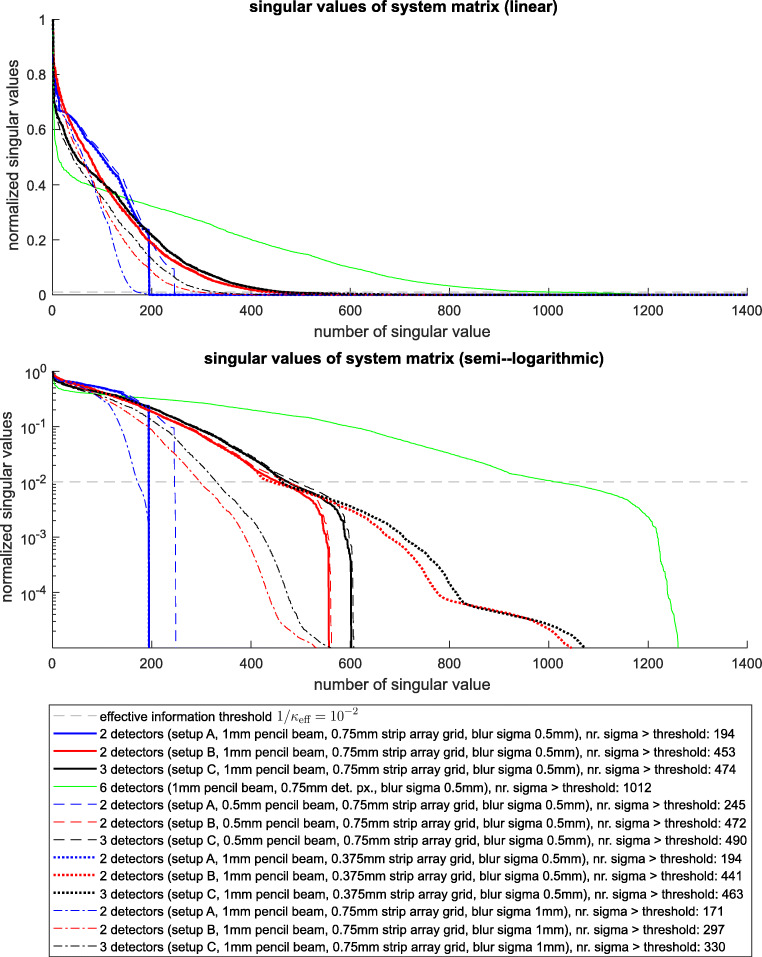
Singular values of the setup A, B, and C and an additional configuration with 6 detectors. Top: representation with linear *y*-scaling, Bottom: representation with logarithmic *y*-scaling. The additional simulation scenarios include as follows: using 6 detectors equiangularly arranged around the MLC (leading to a 2400 × 6750 system matrix) (green line), smaller pencil beam step sizes of 0.5 mm which doubles the pencil beams (leading to 800 × 13500 and 1200 × 13500 system matrices) (dashed lines), reducing the detector pixel size from 0.75 to 0.375 mm (leading to 1600 × 6750 and 2400 × 6750 system matrices) (fat dotted lines), and doubling the blur in the fluence signal from *σ* = 0.5 mm to *σ* = 1 mm (which is not changing the system matrix size relative to the start scenario) (dash-dotted lines)

Based on these system matrices, the singular value distribution is present in Fig. 6. Essentially one needs to focus on the singular values with logarithmic scaling and at which point the values drop under the effective information threshold. The number of singular values above this threshold tells how many useful constraints (information) is present in Matrix *A* about the values $\vec {\beta }$.

A range of scenarios is presented which help interpreting the results. In this plot also the *effective information threshold* is shown. For example, if we assume that the amplitude of the measurement signal of each detector pixel is a 100 times higher than the noise (an already good signal in practice), this corresponds to a $\text {SNR} = 20\log _{10}\left (\frac {A_{\text {signal}}}{A_{\text {noise}}}\right ) \text {dB} = 20\log _{10}(100) \text {dB} = 40 \text {dB}$. If we further allow in the worst case for a 100*%* error in $\vec {\beta }$ (although it typically is much better), then the effective condition number is *κ*_eff_ = 100. In the case, that this estimation seems too risky, also a lower effective condition number, such as *κ*_eff_ = 10, can be chosen, without changing the result of system comparison. The simulations show (most effectively looking at the logarithmic graph at the bottom of Fig. 6):

1. If one uses more detectors, say 6 and arrange them equiangularly around the MLC this generates truly new information (green line) which helps reconstructing the true fluence field. This shows the way of optimal signal reconstruction: if one increases the number of detectors, one can reconstruct the original signal better and better.
2. If one uses smaller pencil beams (0.5-mm steps instead of 1-mm steps, i.e., doubling the number of pencil beams) in order to approximate true fluence fields even better (dashed lines), this essentially does not change information content in these system matrices (although a slight improvement for the setup A is visible).
3. If one reduces the pixel size of the detector (i.e., doubling each detector signal), we see that for setup A, there is no improvement at all and for setup B and C, there is improvement, but at a neglectable information level since this new information is way below the *effective information threshold* corresponding to the condition number 100.
4. If there is twice as much blur in the fluence field, then the total information content for all detector configurations is equivalently reduced.

Based on this overview plot, it is clearly favorable to use setup B compared to setup A, since much more constraints and information are generated by this detector configuration. The introduction of another horizontal projection in setup C compared to setup B shows a rather minor improvement concerning *new information* introduced by this additional detector.

### Determing optimal detection geometry

Since we have a number which allows the comparison of different detector configurations, we can formally optimize detector configurations. Before starting optimization, certain constraints need to be defined, such as *number of detectors*, *degrees of freedom* (e.g., angular position of each detector), and an *inital detector configuration* which will be modified by the degrees of freedom. Furthermore, an *effective information threshold* must be set which should be adjusted to the measurement quality of the observation at hand.

**Fig. 7:**
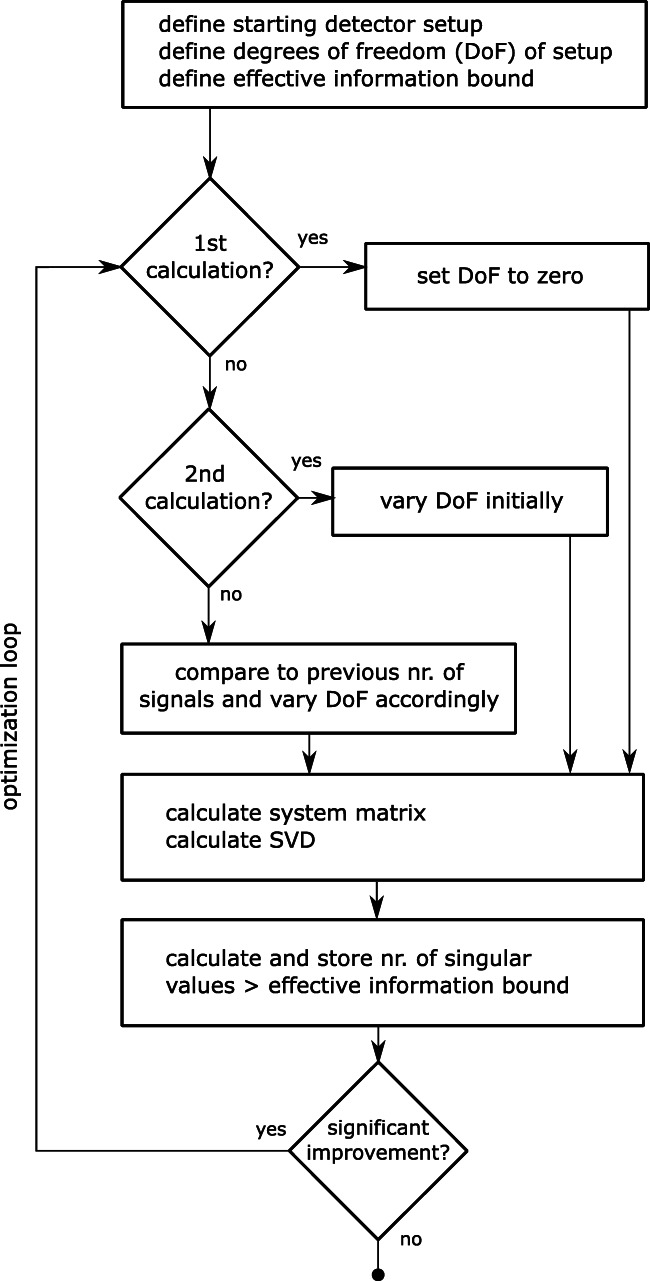
Optimization scheme, which includes the presented system performance estimation and allows for algorithmic maximization of the information contained in the system configuration

After this, an optimization scheme (e.g., see Fig. 7) can be applied. It must be stated that this optimization can be very time consuming, since possibly very large system matrices need to be calculated in a loop.

## Discussion

The singular value decomposition (SVD) and the condition number of a matrix *κ*(*A*) are a mathematical standard toolset in order to judge about the practical/numerical difficulty of inverting a linear equation. Applied to an inversion problem, it summarizes information about how challenging this problem actually is, and not how well a specific inversion algorithm performs. If, e.g., the condition number of a matrix is very large with respect to the involved uncertainties (numerical precision, measurement noise, etc.) of the linear equation, every inversion/reconstruction algorithm will be confronted with this challenging problem. The SVD-based analysis of a system allows measuring how difficult an inversion/reconstruction problem is at its core, and demonstrates a very useful tool for (linearized) inverse problems in general.

In this paper, we utilized the relation between SVD and the condition number in the following way: For competing configurations of an inverse problem, we state that if the difficulty of inversion/reconstruction is less for one configuration than for another, then more useful information is actually acquired and it is preferable. This corresponds to maximizing the number of singular values greater than an *effective information threshold*. The practical difficulty of this approach lies in the modeling and mathematical formulation of a given measurement scenario and an at least approximate linear formulation of the observation equation, as presented. The strength of the approach is, once this modeling worked out, the simple steps in Section 2.2.4 for comparing different observation scenarios can be directly applied without any inverse computations.

It is important to notice that this investigation with SVD of the system matrix essentially only focuses on how much *new information* is generated by *alternative detector configurations* compared to each other. There still is a point to be made that redundant information has its own merit, since this typically increases robustness and reduces dependencies on measurement noise. Furthermore, even if in a specific application it is unclear how to determine the *effective information threshold* exactly, system comparison can be performed well, by using several reasonable thresholds in a given range and compare these systems for all these thresholds simultaneously.

We are not aware of any other method that would perform a similar type of high- or general-level analysis that could be used for the determination of detector/system configurations. That is performing a meta-analysis without the need to use specific radiotherapy beam shapes for specific patient anatomies and specific radiotherapy beam reconstruction algorithms.

**Fig. 8:**
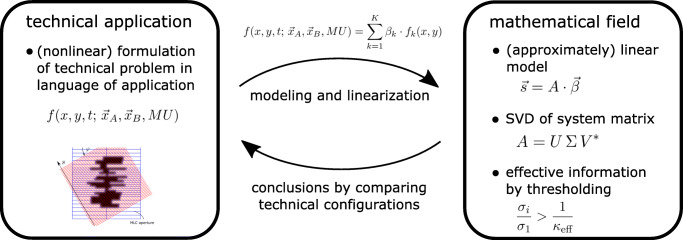
Illustration of the general approach for performing *configuration meta-analysis*

The concrete application of this methodology is to compare detector geometries for multileaf collimator measurements in radiotherapy and it demonstrates that this method can even be applied to not obvious cases and still allows for a solid judgment. In this specific example, the linearization also comes at a cost: In our linear model, we allow for much more 2D fluence fields than would be practically possible, which enormously increases the degrees of freedom from 2 ⋅ 45 + 1 = 91 degrees of freedom (each leaf position + MU) to 6750 degrees of freedom. Still, with this approach, all real 2D fluence fields can be approximated well, and therefore, this investigation is practically useful. It is pointed out that the clinical use of setup C investigated with this singular value analysis is additionally demonstrated for several patient treatment plans for a specific reconstruction approach as presented in [11]. In the schematic Fig. 8 we are summarizing this general modeling approach directly, which may be applied also to different technical applications in the future.

In radiotherapy, other applications of this general approach might be beneficial such as in external beam treatment planing optimization which is not strictly speaking a detection or measurement scenario. However, it can still be expressed in similar terms as follows: clinical parameters of interest (MLC apertures), the dose metric prescribed by the physician (which corresponds to the measurements in the detection design), system matrix (dose calculation model), and configurations (on which the dose calculation model depends). In this application, the modeling, optimization, and analysis of an overall dose delivery to the patient are typically done by a superposition (i.e., linearization) of pencil beams. In this example, optimal beam angles, number of MLC segments per beam angle, or even comparisons between different dose delivery techniques, such as conformal treatment, IMRT, and VMAT, could be investigated as clinically relevant parameters.

Finally, we want to point out that the linearization method presented in this paper may not necessarily be the best way for reconstructing the clinical parameters $\vec {\beta }$, but it provides a quality figure of the system configuration. In specific cases, it is possible (e.g., if more observations *N* are made than there are degrees of freedom *K*) to directly invert the observation equation with a low rank approximation of the system matrix *A*, leading, e.g., to the *pseudo-inverse* [23]. In our specific application, we demonstrate that this investigation via SVD is still possible and useful, even if such an inversion is not possible, since we focus on a highly underdetermined system.

## Conclusion

We have demonstrated a general approach based on the singular values of a system matrix, which allows to compare different system configurations regarding their effective information content. This presentation contains an extension to previous argumentation found in the literature with respect to the definition, meaning, and utility of the information content, cp. [4, 6, 13, 25, 26]. In order to demonstrate the use of this method for a challenging nonlinear example, we compared possible detection configurations for multileaf collimator control measurements in external beam therapy in radiotherapy. Based on this, the application of this method is demonstrated, including the linearization of the nonlinear model, and the best system configuration is determined.

## Figures and Tables

**Fig. 9:**
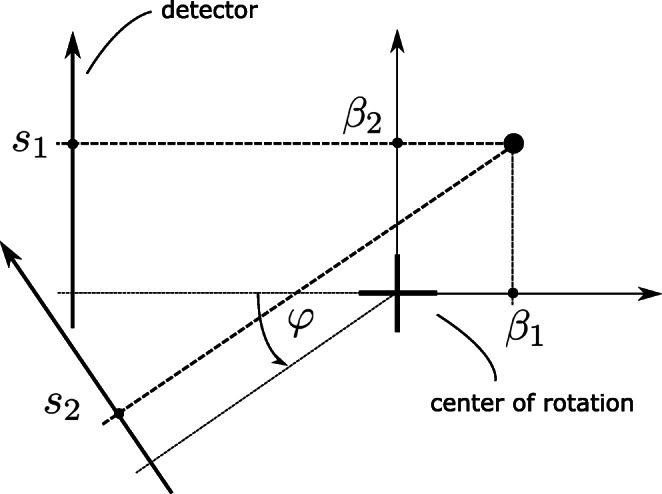
Explanatory example: The 2D point coordinates (*β*_1_,*β*_2_) need to be reconstructed with a given detector configuration of two detectors which are rotated relatively to each other by *φ*, leading to detection signals *s*_1_ and *s*_2_. Different angles *φ* correspond to different detector configurations

## Appendix 1. Explanatory example

In order to present the strength of this approach, we are briefly presenting the following measurement setup taken from reference [10], presented in Fig. 9.

In this example, we want to measure the 2D point coordinates (*β*_1_,*β*_2_). Furthermore, we have a rotating detector which measures the parallel beam projection of the 2D point coordinate in the detector coordinate *s*. First, there is a horizontal measurement performed and *s*_1_ is measured, then the detector is rotated by *φ* and the second detector point *s*_2_ is measured. The configuration parameter *φ* simply corresponds to different detector configurations. It is clear, if there is no noise in the measurements *s*_1_ and *s*_2_ and we use *φ*≠ 0 the coordinates (*β*_1_,*β*_2_) can be reconstructed directly by just inverting this linear system of equations. On the other side, it is clear that using *φ* ≈ 0 will be worse than *φ* ≈ 90^∘^ if there is measurement noise. In order to quantify this, we want to apply the proposed process:

Step 1: This leads to the linear observation/detection model

$$ \vec{s} = \left( \begin{array}{c}s_{1}\\s_{2} \end{array}\right) = \left( \begin{array}{cc} 0 & 1 \\ -\sin(\varphi) & \cos(\varphi) \end{array}\right)\cdot \left( \begin{array}{c}\beta_{1}\\\beta_{2} \end{array}\right) = A\cdot\vec{\beta} . $$

Step 2: We define the expected level of relative measurement error, e.g. $\frac {\|\vec {\varepsilon }\|}{\|\vec {s}\|}=10^{-2}$.

Step 3: We define a target relative solution error, e.g., $\frac {\| {\Delta } \vec {\beta } \|}{\| \vec {\beta } \|}=10^{-1}$

Step 4: This leads to the condition number of the sharp bound *κ*_eff_ = 10.

Step 5: We calculate the singular values of *A*, which are

$$ \sigma_{1} = \sqrt{1+\cos(\varphi)}, \sigma_{2} = \sqrt{1-\cos(\varphi)} $$

In this example, the qualitatively most important question is simply, whether the second measurement contains enough information for the given relative errors in order to invert the linear system of equations or not. This means, we need the second singular value to be above the effective information threshold

$$ \frac{\sigma_{2}}{\sigma_{1}} > \frac{1}{\kappa_{\text{eff}}} \Rightarrow \sqrt{\frac{1-\cos(\varphi)}{1+\cos(\varphi)}} > \frac{1}{\kappa_{\text{eff}}} . $$

This leads to the solutions in [0^∘^, 180^∘^]:

$$ \begin{array}{@{}rcl@{}} \varphi &\in& \left] \arccos\left( \frac{\kappa_{\text{eff}}^{2}-1}{\kappa_{\text{eff}}^{2}+1}\right),180^{\circ} \right. \\ &&\left. -\arccos\left( \frac{\kappa_{\text{eff}}^{2}-1}{\kappa_{\text{eff}}^{2}+1}\right) \right[ \approx \left]11.43^{\circ},168.57^{\circ}\right[ . \end{array} $$

Step 6: The comparison of different detector configurations then is simply: if *φ* is in this calculated interval, then in general, we have enough information to invert the linear system of equations with the defined target relative solution error. This is a qualitative step. Inside this interval for *φ* one could further distinguish between all configurations, which leads to the optimum of *φ* = 90^∘^ corresponding to the highest possible ratio $\frac {\sigma _{2}}{\sigma _{1}}=1$.

Since we compare system configurations with a large number of singular values in practice, we typically end up with a different number of singular values above the effective information threshold, leading to a well-defined quantity for comparison. Only if two configurations have roughly the same number, it makes sense to take the shape of the distribution of the singular values into account.

Furthermore, we are demonstrating in the Appendix ?? that the relative error inequality bound is sharp, i.e., this means for specific measurement $\vec {s}$ and noise configurations $\vec {\varepsilon }$, exactly an equality occurs. We want to illustrate this in this explanatory example. If *φ*≠ 0, we can directly solve this equation system with

$$ \begin{array}{@{}rcl@{}} \vec{\beta} &=& \left( \begin{array}{c}\beta_{1}\\\beta_{2} \end{array}\right) = \left( \begin{array}{c}\frac{\cos(\varphi) s_{1}-s_{2}}{\sin(\varphi)}\\ s_{1} \end{array}\right), \\ {\Delta}\vec{\beta} &=& \left( \begin{array}{c}{\Delta} \beta_{1}\\{\Delta} \beta_{2} \end{array}\right) = \left( \begin{array}{c}\frac{\cos(\varphi) \varepsilon_{1} - \varepsilon_{2}}{\sin(\varphi)}\\ \varepsilon_{1} \end{array}\right) . \end{array} $$

On the other side, we have $\kappa (A) = \sqrt {\frac {1+\cos \limits (\varphi )}{1-\cos \limits (\varphi )}}$ and of course $\vec {s} = (s_{1},s_{2})^{T}$ and $\vec {\varepsilon }= (\varepsilon _{1},\varepsilon _{2})^{T}$. Then, the relative error inequality leads for all $\vec {s}$ and $\vec {\varepsilon }$ to

$$ \begin{array}{@{}rcl@{}} \frac{\| {\Delta} \vec{\beta} \|}{\| \vec{\beta} \|} \!&\leq&\! \kappa(A)\cdot\frac{\| \vec{\varepsilon} \|}{\| \vec{s} \|} \!\Leftrightarrow\! \sqrt{ \frac{ {\varepsilon_{1}^{2}} \sin^{2}(\varphi) + (\cos(\varphi) \varepsilon_{1} - \varepsilon_{2})^{2} }{ {s_{1}^{2}} \sin^{2}(\varphi) + (\cos(\varphi) s_{1} - s_{2})^{2} }} \\ &\leq& \sqrt{\frac{1+\cos(\varphi)}{1-\cos(\varphi)}} \sqrt{\frac{{\varepsilon_{1}^{2}} + {\varepsilon_{2}^{2}}}{{s_{1}^{2}}+{s_{2}^{2}}}} . \end{array} $$

If we find a configuration of $\vec {s}$ and $\vec {\varepsilon }$ leading to the equality of left and right hand side, than we see this bound is sharp and cannot be improved.

Utilizing the left singular vectors of *A* (see the Appendix ?? for the general case), we get these vectors *pointing to their worst directions* with $\vec {s}=\lambda \cdot (1,1)^{T}$ and $\vec {\varepsilon }=\mu \cdot (1,-1)^{T}$, with arbitrary constants $\lambda ,\mu \in \mathbb {R}$. By inserting them into the inequality, we get on both sides $\frac {\mu }{\lambda }\sqrt {\frac {1+\cos \limits (\varphi )}{1-\cos \limits (\varphi )}}$, i.e., the equality holds and the bound is sharp.

On the other hand, we realize that a very specific configuration is necessary that the equality holds, i.e., *s*_1_ = *s*_2_ and *ε*_1_ = −*ε*_2_ (remember, the latter is random noise). In total, this demonstrates that the relative error bound is sharp, but in practice, equality is probably never reached. In cases with much higher dimensionality of $\vec {\beta }$ and $\vec {s}$, such as in the application presented next, the likelihood of achieving equality decreases further since the configurations for the worst case are getting more specific.

## Appendix : 2. Worst case discussion of the relative error bound

In order to actually fulfill the equality of the relative error upper bound, $\vec {\varepsilon }$ and $\vec {s}$ would need to simultaneously point in their *worst directions*. In order to see this, we start with the full linear system including the errors:

$$ A\cdot\left( \vec{\beta}+{\Delta}\vec{\beta}\right) = \vec{s} + \vec{\varepsilon} . $$

By applying the SVD (here in its *compact form*, i.e., *r* representing the rank of *A*, $U\in \mathbb {R}^{N\times r}$, $V\in \mathbb {R}^{K\times r}$ and ${\Sigma }\in \mathbb {R}^{r\times r}$, and *U* and *V* *semi–unitary*, i.e., *U*^∗^⋅ *U* = *V*^∗^⋅ *V* = *I*_*r*×*r*_), we get

$$ \Leftrightarrow U\cdot{\Sigma}\cdot V^{*} \cdot\left( \vec{\beta}+{\Delta}\vec{\beta}\right) = \vec{s} + \vec{\varepsilon} . $$

with *U* containing the first *r* left singular vectors as columns, Σ is the diagonal matrix with decreasing order of the *r* singular values and *V* containing the first *r* right singular vectors as columns. Due to the *semi–unitarity* of *U* we can rewrite this to

$$ \Leftrightarrow {\Sigma}\cdot V^{*} \cdot\left( \vec{\beta}+{\Delta}\vec{\beta}\right) = U^{*}\cdot\left( \vec{s} + \vec{\varepsilon}\right) . $$

If we specifically choose $\vec {s}$ to be a scaling of the first left singular vector, i.e., $\vec {s} = \lambda \vec {u}_{1}$, and $\vec {\varepsilon }$ to be a scaling of the *r* th left singular vector, i.e., $\vec {\varepsilon } = \mu \vec {u}_{r}$ ($\lambda ,\mu \in \mathbb {R}$), we get

$$ \begin{array}{@{}rcl@{}} &\Rightarrow & {\Sigma}\cdot V^{*} \cdot\left( \vec{\beta}+{\Delta}\vec{\beta}\right) = \left( \begin{array}{c} \lambda\\0\\\vdots\\0 \end{array}\right) + \left( \begin{array}{c} 0\\\vdots\\0\\\mu \end{array}\right)\\ &\Leftrightarrow & V^{*} \cdot\left( \vec{\beta}+{\Delta}\vec{\beta}\right) = \left( \begin{array}{c} \frac{\lambda}{\sigma_{1}}\\0\\\vdots\\0 \end{array}\right) + \left( \begin{array}{c} 0\\\vdots\\0\\ \frac{\mu}{\sigma_{r}} \end{array}\right) . \end{array} $$

An obvious solution to this is

$$ \vec{\beta}+{\Delta}\vec{\beta} = \frac{\lambda}{\sigma_{1}}\cdot \vec{v}_{1} + \frac{\mu}{\sigma_{r}}\cdot \vec{v}_{r} , $$

with the right singular vectors $\vec {v}_{1}$ and $\vec {v}_{r}$. When inserting these $\vec {\beta }$, ${\Delta }\vec {\beta }$, $\vec {s}$, and $\vec {\varepsilon }$ into the relative error upper bound, one gets the worst case with equality. In this solution, one can also directly interpret the worst case interaction between measurement quality and the system configuration of the relative error bound: If we have a SNR of the measurement, say 100, this can be interpreted as $\frac {\lambda }{\mu }=100$, then in order to allow for a maximum error ${\Delta }\vec {\beta }$ to be in the range of $\vec {\beta }$, one can only allow for $\frac {\sigma _{1}}{\sigma _{r}}=100=\kappa (A)$. For lower condition numbers *κ*(*A*), we get $\frac {\lambda }{\sigma _{1}} > \frac {\mu }{\sigma _{r}}$, leading to higher quality solutions in this worst case.

From a practical perspective, in order to actually run into this worst case region, $\vec {s}$ and $\vec {\varepsilon }$ simultaneously would need to be linear combinations of very specific directions, such for $\vec {s}$ in the span of vectors with the highest singular values $\text {span}\left (\vec {u}_{1},\dots \right )$, and $\vec {\varepsilon }$ in the span of vectors with the lowest singular values $\text {span}\left (\dots ,\vec {u}_{r}\right )$. This means, if $\vec {\varepsilon }$ is a random detector measurement error, expected to be independently distributed for each coordinate, this could be assigned to a certain low probability. Therefore, practically in many cases, one can expect much better results than what the relative error upper bound would suggest. See also the explanatory example, where this argument is directly demonstrated.

## References

[1] Albert S, Brivio D, Aldelaijan S, Sajo E, Hesser J, Zygmanski P (2020). Towards customizable thin-panel low-Z detector arrays: electrode design for increased spatial resolution ion chamber arrays. Phys Med Biol.

[2] Katharina P (2014). Aschenbrenner Master’s Thesis: 2D MLC leaf position measurement from projection data.

[3] Brivio D, Albert S, Gagne MP, Freund E, Sajo E, Zygmanski P (2020) Nanoporous aerogel-based periodic high-energy electron current x-ray sensors. United Kingdom N. Web. 10.1088/1361-6463/ab83c010.1088/1361-6463/ab83c0

[4] Culver JP, Ntziachristos V, Holboke MJ, Yodh AG (2001). Optimization of optode arrangements for diffuse optical tomography: a singular-value analysis, Opt. Lett..

[5] Damen AA, van der Kam J (1982). The use of the singular value decomposition in electrocardiography. Med Biol Eng Comput.

[6] Graves EE, Culver JP, Ripoll J, Weissleder R, Ntziachristos V (2004). Singular-value analysis and optimization of experimental parameters in fluorescence molecular tomography. J Opt Soc Am A.

[7] Fujimoto K, Scherpen JMA (2008) Singular value analysis and balanced realizations for nonlinear systems. In: Rommes J, Schilders WHA, Vorst HAVD (eds) Model order reduction: theory, research aspects and applications. (13 ed., pp 251-272) (Mathematics in Industry; No. 13), pp 251–271

[8] Han Z, Hacker F, Killoran J, Kukluk J, Aizer A, Zygmanski P (2019). Optimization of MLC parameters for TPS calculation and dosimetric verification: application to single isocenter radiosurgery of multiple brain lesions using VMAT. Biomed Phys Eng Express.

[9] Hirashima H, Nakamura M, Miyabe Y, Uto M, Nakamura K, Mizowaki T (2018). Monitoring of mechanical errors and their dosimetric impact throughout the course of non-coplanar continuous volumetric-modulated arc therapy. Radiat Oncol.

[10] Hoegele W, Loeschel R, Dobler B, Koelbl O, Zygmanski P (2013) Bayesian estimation applied to stochastic localization with constraints due to interfaces and boundaries. Mathematical Problems in Engineering

[11] Hoegele W, Zygmanski P (2022). Strip detector array (SDA) for beam monitoring in radiotherapy: reconstruction of MLC parameters from multiple projections of flux. Biomed Phys Eng Express.

[12] Kyprianou IS, Badano A, Gallas BD, Myers KJ (2008). Singular value description of a digital radiographic detector: theory and measurements. Med Phys.

[13] Lasser T, Ntziachristos V (2007). Optimization of 360 degrees projection fluorescence molecular tomography. Med Image Anal.

[14] Leblond F, Kenneth M, Pogue BW, Tichauer (2010). Singular value decomposition metrics show limitations of detector design in diffuse fluorescence tomography. Biomed Opt Express.

[15] Lim SB, Zwan BJ, Lee D, Greer PB, Lovelock DM (2021). A novel quality assurance procedure for trajectory log validation using phantom-less real-time latency corrected EPID images. J Appl Clin Med Phys.

[16] Lorenz F, Nalichowski A, Rosca F, Kung J, Wenz F, Zygmanski P (2007). Spatial dependence of MLC transmission in IMRT delivery. Phys Med Biol.

[17] Lorenz F, Killoran JH, Wenz F, Zygmanski P (2007). An independent dose calculation algorithm for MLC-based stereotactic radiotherapy. Med Phys.

[18] Lorenz F, Nalichowski A, Rosca F, Killoran J, Wenz F, Zygmanski P (2008). An independent dose calculation algorithm for MLC-based radiotherapy including the spatial dependence of MLC transmission. Phys Med Biol.

[19] Mayles P, Nahum A, Rosenwald J-C (eds) (2007) Handbook of radiotherapy physics: theory and practice. Taylor & Francis Group, Milton Park

[20] Poppe B, Thieke C, Beyer D, Kollhoff R, Djouguela A, Rühmann A, Willborn KC, Harder D (2006). DAVID–a translucent multi-wire transmission ionization chamber for in vivo verification of IMRT and conformal irradiation techniques. Phys Med Biol.

[21] Renaud J, Muir B (2022). Assessing the accuracy of electronic portal imaging device (EPID)-based dosimetry: i. Quantities influencing long-term stability. Med Phys.

[22] Rosca F, Zygmanski P (2008). An EPID response calculation algorithm using spatial beam characteristics of primary, head scattered and MLC transmitted radiation. Med Phys.

[23] Strang G (2016). Introduction to linear algebra.

[24] Somai V, Legrady D, Tolnai G (2018). Singular value decomposition analysis of back projection operator of maximum likelihood expectation maximization PET image reconstruction. Radiol Oncol.

[25] Wang D, Liu X, Liu F, Bai J (2010). Full-angle fluorescence diffuse optical tomography with spatially coded parallel excitation. IEEE Trans Inf Technol Biomed.

[26] Heng X, Dehghani H, Pogue BW, Springett R, Paulsen KD, Dunn JF (2003) Near-infrared imaging in the small animal brain: optimization of fiber positions, J Biomed Opt 8(1) 10.1117/1.1528597 12542386

[27] Zygmanski P, Halvorsen P (2021). Routine pretreatment patient-specific IMRT QA (PS-IMRT-QA) should be discontinued and replaced with a real-time on-board beam monitoring system (BMS). Med Phys.

